# Enhanced Fear Expression in a Psychopathological Mouse Model of Trait Anxiety: Pharmacological Interventions

**DOI:** 10.1371/journal.pone.0016849

**Published:** 2011-02-28

**Authors:** Simone B. Sartori, Markus Hauschild, Mirjam Bunck, Stefano Gaburro, Rainer Landgraf, Nicolas Singewald

**Affiliations:** 1 Department of Pharmacology and Toxicology, Center for Molecular Biosciences Innsbruck (CMBI) and Institute of Pharmacy, University of Innsbruck, Innsbruck, Austria; 2 Department of Behavioral Neuroendocrinology, Max Planck Institute of Psychiatry, Munich, Germany; University of Chicago, United States of America

## Abstract

The propensity to develop an anxiety disorder is thought to be determined by genetic and environmental factors. Here we investigated the relationship between a genetic predisposition to trait anxiety and experience-based learned fear in a psychopathological mouse model. Male CD-1 mice selectively bred for either high (HAB), or normal (NAB) anxiety-related behaviour on the elevated plus maze were subjected to classical fear conditioning. During conditioning both mouse lines showed increased fear responses as assessed by freezing behaviour. However, 24 h later, HAB mice displayed more pronounced conditioned responses to both a contextual or cued stimulus when compared with NAB mice. Interestingly, 6 h and already 1 h after fear conditioning, freezing levels were high in HAB mice but not in NAB mice. These results suggest that trait anxiety determines stronger fear memory and/or a weaker ability to inhibit fear responses in the HAB line. The enhanced fear response of HAB mice was attenuated by treatment with either the α_2,3,5_-subunit selective benzodiazepine partial agonist L-838,417, corticosterone or the selective neurokinin-1 receptor antagonist L-822,429. Overall, the HAB mouse line may represent an interesting model (i) for identifying biological factors underlying misguided conditioned fear responses and (ii) for studying novel anxiolytic pharmacotherapies for patients with fear-associated disorders, including post-traumatic stress disorder and phobias.

## Introduction

Fear is a phasic, apprehensive arousal to an explicit threat of an aversive stimulus dissociable from a long-term state of anxiety [1]. Fear and anxiety are part of a universal survival strategy ensuring adaptive responses to a threat, returning to baseline activity once it has passed [2]. In some individuals, though not all, the experience of an aversive situation that elicits extreme fear may cause a prolonged and/or inappropriate response which even persists after withdrawal of the stimulus. Such subjects develop a pathological form of anxiety including post-traumatic stress disorder (PTSD) or phobia [3], [4], [5]. What it is that determines either resilience or the propensity to developing an anxiety disorder is thought to result from a combination of biological factors that are heritable and diverse learning experiences gained in early life [6], [7]. For example, trait anxiety is considered to be a major risk factor for anxiety disorders [8] as well as depression [9].

Fear and anxiety have some parallels as they seem to be genetically linked ([10], [11], [12], [13], [14]; but see [15], [16]), and also as they share common neurocircuitries and brain areas, including the prefrontal cortex, the hippocampus and the amygdala (e.g. for review see [1], [17], [18], [19]). In addition, the same pharmacotherapies, including the prototypical anxiolytics benzodiazepines (BZD), are effective in patients suffering from diverse forms of anxiety disorders, whether or not they are the result of conditioning processes [20], [21], [22].

Regarding pathological anxiety, some of the major challenges in neuroscience involve developing tools for the identification of vulnerable subjects, preventing the onset of an anxiety disorder and, once it is established, discovering the best treatment targets and strategies. The present study was therefore aimed at clarifying the impact of high innate anxiety on experience-based learned fear by characterizing contextual and cued conditioned fear responses in two mouse lines selectively bred for either high (HAB) or normal (NAB) anxiety-related behaviour [23]. These paradigms differentially involving the amygdala and hippocampus in animals and humans [24], [25] were chosen because conditioning processes occur in the aetiology of many anxiety disorders, including phobias and PTSD [26], [27]. Furthermore, we investigated whether any of the following treatments were able to reduce high conditioned fear responses in this psychopathological model: the novel anxiolytic L-822,429, a selective neurokinin-1 (NK1) receptor antagonist [28], [29], corticosterone (CORT), which has been shown to inhibit the retrieval of particularly emotionally arousing information [30], [31], [32], and the α_2,3,5_-subunit selective BZD partial agonist L-838,417 [33].

## Results

### Flinch/jump test

No differences were revealed between HAB (n = 4) and NAB (n = 4) mice in their threshold for flinching (HAB: 0.33±0.01 mA; NAB: 0.28±0.03 mA; U_6,8_ = −0.624, *p* = 0.686), jumping (HAB: 0.49±0.02 mA; NAB: 0.44±0.03 mA; U_4.5,8_ = −1.042, *p* = 0.343) or vocalizing (HAB: 0.60±0.02 mA; NAB: 0.52±0.02 mA; U_2.5,8_ = −1.648, *p* = 0.114) indicating that foot shocks with 0.7 mA were sufficient to induce aversive responses in both lines and, thus, may be applied as unconditioned stimulus (US) for fear conditioning.

### Fear conditioning

**Figure 1:**
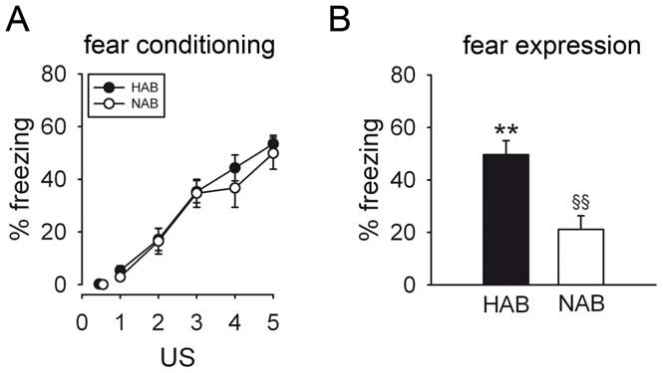
Contextual conditioned fear in HAB and NAB mice. Although freezing behaviour (assessed during each 2-min period post US presentation) increased in HAB and NAB animals in response to 5 US presentations to the same extent (A), indicating comparable fear conditioning of both lines, HAB mice displayed significantly higher fear responses than NAB mice when exposed to the conditioning context for 3 min 24 h later (B). Data are presented as means ± SEM. *n* = 9 per line. **p<0.01 HAB vs. NAB, ^§§^ *p*<0.01 fear expression vs. last US presentation.

**Figure 2:**
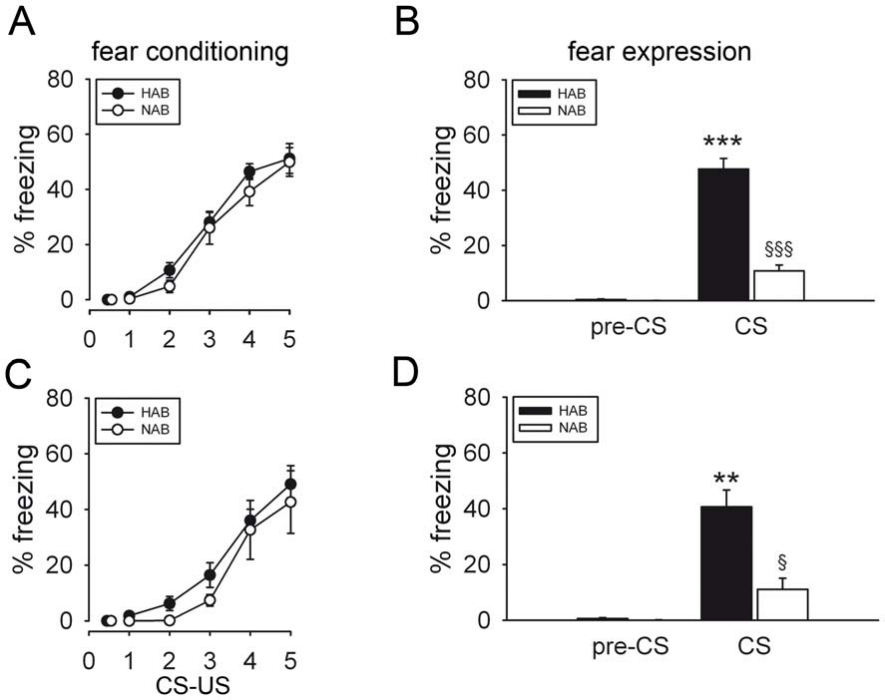
Cued conditioned fear in HAB and NAB mice during the light (A, B) and dark (C, D) phases of the circadian cycle. Both lines showed the same increasing conditioned responses to the CS-US presentations, as indicated by freezing levels during CS presentation (A, C). In contrast, compared with NAB mice, HAB mice showed more pronounced freezing levels in response to the CS only during fear expression performed 24 h later (B, D). Note that freezing levels prior CS presentation (pre-CS) were negligible indicating that there was no contextual fear component (B, D). Data are presented as means ± SEM. *n* = 7–11 per line. **p<0.01, ***p<0.001 HAB vs. NAB; ^§^ *p*<0.05, ^§§§^ *p*<0.001 fear expression vs. last CS-US pairing.

When animals were placed into the conditioning chamber for fear conditioning (Figures 1A and 2A) or into an empty mouse cage for cued fear expression (Figures 2B and D), the freezing behaviour was negligible in both HAB and NAB lines (<3%) indicating that there was no contextual fear component in cued auditory fear expression. No statistically significant difference between lines in baseline freezing to both contexts was observed.

#### Contextual fear conditioning

Upon repeated US presentations conditioned responses as indicated by freezing behaviour increased to the same extent in both the HAB and NAB lines (pairing effect: *F*_4,68_ = 53.482, *p*<0.001; line effect: *F*_1,17_ = 0.439, *p* = 0.516; line x pairing interaction: *F*_4,68_ = 0.304, *p* = 0.874), reaching a maximum of approximately 54% freezing (Figure 1A). 24 h later, when animals were re-exposed to the conditioning context for 3 min, freezing levels were elevated in HAB mice compared with NAB mice (*t* = 3.793, df = 17, *p* = 0.002; Figure 1B). Compared with the period post the last US presentation on the conditioning day, freezing levels were reduced in NAB mice (*t* = 3.637, df = 8, *p* = 0.007) during fear expression, but not in HAB mice (*t* = 0.672, df = 9, *p* = 0.518; Figure 1B).

#### Cued fear conditioning

Irrespective of whether conditioning was performed during the light phase or the dark phase of the cycle, both HAB and NAB mice acquired cued conditioned fear as indicated by an increase in freezing levels of up to 50–60% within five pairings of a 2-min tone (conditioned stimulus; CS) co-terminating with the US (pairing effect during light phase: *F*_4,72_ = 75.011, *p*<0.001; during dark phase: *F*_4,52_ = 31.657, *p*<0.001; Figures 2A and 2C). Two-way ANOVA with repeated measures revealed no significant line effect (light phase: *F*_1,18_ = 0.955, *p* = 0.342; dark phase: *F*_1,13_ = 0.676, *p* = 0.426) or line x pairing interaction (light phase: *F*_4,72_ = 0.345, *p* = 0.847; dark phase: *F*_4,52_ = 0.230, *p* = 0.883) during either the light or the dark phase. 24 h later, expression of cued conditioned fear by presenting 3 CSs was tested in animals of both lines. HAB mice differed greatly from NAB mice in their fear responses to the CS during both the light phase (*t* = 8.851, df = 18, *p*<0.001; Figure 2B) and the dark phase (*t* = 3.938, df = 13, *p* = 0.002; Figure 2D) of the cycle, with HAB mice displaying more pronounced freezing levels than NAB mice. While NAB mice showed significantly lower freezing levels during the fear-expression test than during the last CS-US pairing of the conditioning session (light phase: *t* = 6.091, df = 10, *p*<0.001; dark phase: *t* = 3.588, df = 6, *p* = 0.012; Figure 2), freezing levels between these two testing periods were found similar in HAB mice (light phase: *t* = 0.674, df = 8, *p* = 0.520; dark phase: *t* = 1.850, df = 7, *p* = 0.107; Figure 2). The circadian cycle did not affect the percentages of freezing displayed by the two lines at the end of the fear conditioning (line effect: *F*_1,31_ = 1.616, *p* = 0.213; line x pairing x cycle interaction: F_4,124_ = 0.355, *p* = 0.840) or during fear expression (line effect: F_1,31_ = 0.701, *p* = 0.409; line x cycle interaction: F_1,31_ = 0.841, *p* = 0.366). During the dark phase unconditioned HAB and NAB mice (i.e. exposed to the CS without receiving any CS-US pairing) displayed very low freezing levels in response to the CS at the end of the fear conditioning (HAB: 0.24±0.24%; NAB: 0%) as well as during the fear-expression test (HAB: 0.93±0.34%; NAB: 0.62±0.30%). Conditioned and unconditioned animals differed significantly in fear responses during both sessions (pairing x CS interaction during fear conditioning: *F*_4,112_ = 28.085, *p*<0.001; line x CS interaction during fear expression: *F*_1,27_ = 16.250, *p*<0.001).

**Figure 3:**
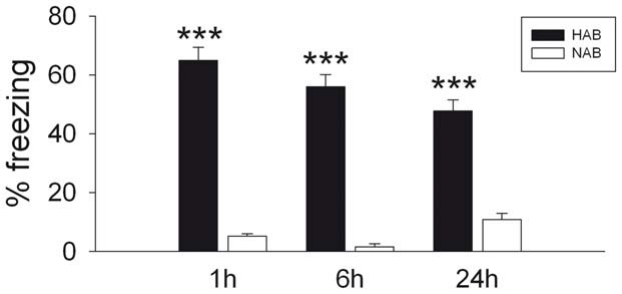
Short- and long-term recall of cued conditioned fear in HAB and NAB mice. CS only was presented to separate HAB and NAB groups 1 h, 6 h (short-term) or 24 h (long-term) after cued fear conditioning. Irrespective of consolidation period, HAB mice showed pronounced freezing during the fear expression test, but not NAB mice. Data are presented as means ± SEM. *n* = 6–8 per experimental group. ***p<0.001 HAB vs. NAB.

In all experiments performed to investigate short-term recall of cued conditioned fear, HAB and NAB animals were conditioned within five CS-US pairings to the same extent (data not shown). As with the 24 h period, expression of cued conditioned fear differed greatly between HAB and NAB mice 1 h (*t* = 15.220, df = 12, *p*<0.001) and 6 h (*t* = 12.825, df = 12, *p*<0.001) after the fear conditioning (Figure 3). There was no significant effect of testing period on fear-expression levels (*F*_2,42_ = 2.475, *p* = 0.096; Figure 3).

#### Pharmacological modulation of learned cued fear in HAB mice

**Figure 4:**
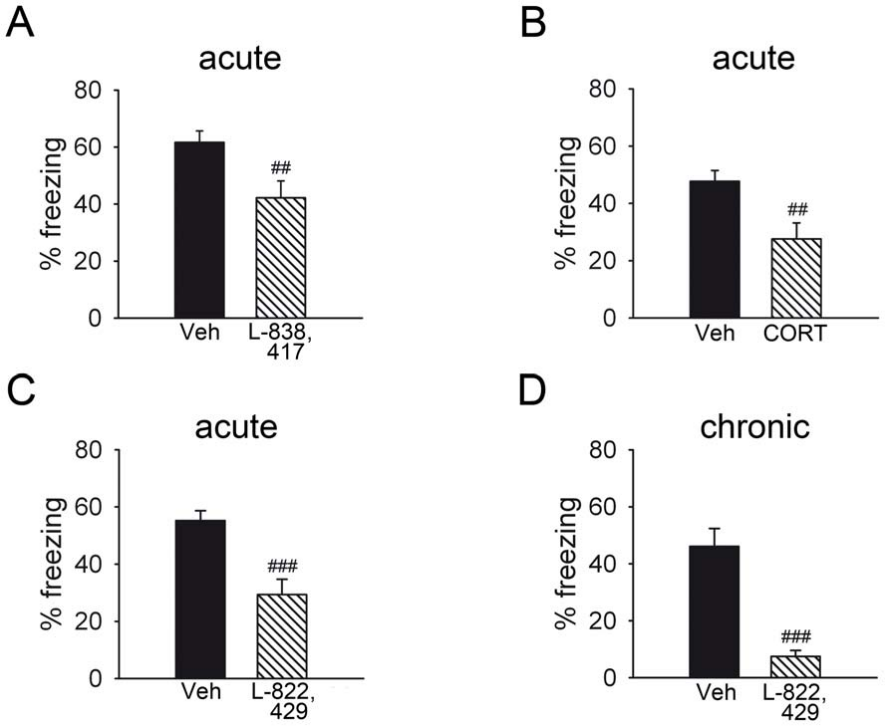
Pharmacological modulation of learned cued fear in HAB mice. An acute application of either the α_2,3,5_-subunit selective benzodiazepine partial agonist L-838,417 (1 mg/kg; A), corticosterone (CORT; 10 mg/kg; B) or the selective NK1 receptor antagonist L-822,429 (30 mg/kg; C) 30 min prior to testing reduced fear expression as indicated by the percentage of freezing displayed by HAB mice during fear expression. Chronic treatment with L-822,429 (30 mg/kg/day; D) attenuated CS-induced freezing levels even more. Data are presented as means ± SEM. *n* = 7–10 per experimental group. ^##^ *p*<0.01, ^###^ *p*<0.001 drug vs. vehicle.

Next we tested the possibility to attenuate the higher fear expression of HAB mice by pharmacotherapeutic interventions using either the α_2,3,5_-subunit selective BZD partial agonist L-838,417, CORT or the selective NK1 receptor antagonist L-822,429. Indeed, all three treatments were effective, though to a different extent (Figure 4). Compared with vehicle treatment, acute application of L-838,417 caused a reduction in freezing levels of 20% in HAB mice (*t* = 3.275, df = 13, *p* = 0.006; Figure 4A). Similarly, HAB mice pre-treated with CORT displayed 20% lower freezing levels than vehicle-treated controls (*t* = 2.893, df = 17, *p*<0.010; Figure 4B) while the NK1 receptor antagonist L-822,429 reduced freezing levels by 26% (*t* = 4.055, df = 18, *p*<0.001; Figure 4C). In addition, HAB mice chronically treated with the selective NK1 receptor antagonist L-822,429 displayed 40% less freezing compared with untreated HAB mice (*t* = 6.194, df = 13, *p*<0.001; Figure 4D). However, chronic treatment with the NK1 receptor antagonist did not affect cued fear conditioning (treatment effect: *F*_1,12_ = 1.055, *p* = 0.325; treatment x pairing interaction: *F*_4,48_ = 2.020, *p* = 0.107). The distance travelled, monitored during the first 2-min habituation period before the onset of the CS in HAB mice was not affected by acute treatment with either L-838,417 (vehicle: 436±49 cm; drug: 307±57 cm; *t* = 1.699, df = 13, *p* = 0.113), CORT (vehicle: 498±88 cm; drug: 415±55 cm; *t* = 0.817, df = 17, *p* = 0.425), or L-822,429 (vehicle: 552±85 cm; drug: 397±77 cm; *t* = 1.357, df = 18, *p* = 0.192).

## Discussion

Using classical Pavlovian fear-conditioning paradigms, we have demonstrated that a mouse line characterized by extremely high innate anxiety-related behaviour (HAB) expressed more contextual- and cue-dependent fear relative to NAB controls. In addition, we have shown for the first time that the high level of fear responses exhibited by HAB animals could be reduced by treatment with either the subtype selective BZD agonist L-838,417, the NK1 receptor antagonist L-822,429, or CORT.

There have been various attempts to link learned fear with innate anxiety as a vulnerability factor for the development of anxiety disorders using behaviourally selected rodents or recombinant strains, including the Roman Low-Avoidance rats [11], [12], low open arm rats [34], high fear reactivity rats [10], mice selected for high levels of fear conditioning [13], and recombinant inbred mice [15]. In these models stronger fear conditioning coincides with an increased level of fear expression. On the other hand, fear conditioning and fear expression are not altered in HAB rats [35], or in low fear recovery rats [10], whereas the extinction of their learned (cued) fear memories is impaired; an investigation of extinction of learned fear in the HAB and NAB lines, however, was not within the scope of the present study, although a parallel study (Yen et al., submitted) does examine this issue.

In contrast to these previously described models [10], [11], [13], [15], [34], [35], we report here similar behavioural responses during fear conditioning, but different fear expression in response to the CS by the HAB and NAB lines. The finding of similar fear conditioning in HAB and NAB mice using the present protocol suggests that selection for high trait anxiety does not necessarily result in altered CS-US association in HAB mice. A similar observation in humans displaying individual differences in trait anxiety levels supports this result [36]. It remains to be tested whether HAB and NAB mice would differ in fear acquisition when using a weaker conditioning protocol, such as applying a foot shock just above the flinching threshold (e.g. 0.35 mA).

During fear conditioning, final freezing levels of up to 50% were reached in HAB and NAB mice. Interestingly, the freezing levels we observed in NAB mice at the end of the conditioning session are higher than those previously reported in the CD-1 strain, which range between 2% [37] and 40% [38] freezing. At this stage it has to be emphasized that NAB mice do not represent a normal cohort of CD-1 mice in terms of variability in inborn anxiety, as the selective breeding for mice spending approximately 35% of their time on the open arm of an elevated plus maze [23], [39] resulted in the clustering of an anxiety-related phenotype around the strain mean rather than around the whole anxiety spectrum typical of outbred CD-1 mice [40]. Accordingly, it may well be that in NAB mice fear responses gathered around the mean freezing levels usually displayed by CD-1 mice during fear conditioning. Indeed, when we used the present paradigm on unselected CD-1 mice purchased from a commercial supplier instead of NAB mice, we observed mean freezing levels of approximately 50%, with individual freezing levels ranging from 2% to 80% at the end of the conditioning session (data unpublished).

24 h after fear conditioning animals were tested for their ability to express learned fear. NAB mice showed little freezing behaviour (around 15%), irrespective of whether the CS was a cue or the context. The low freezing levels of NAB mice during fear expression are comparable to those of unselected CD-1 mice observed at the laboratory of our collaborator (Yen et al., submitted) and at our laboratory (data unpublished), as well as to those levels reported previously [37], [38], [41]. These data suggest that NAB mice show normal, strain (CD-1)-specific fear expression 24 h after fear conditioning. In contrast to NAB mice, HAB mice displayed pronounced freezing responses to the CS suggesting that a high anxiety trait may trigger increased fear expression. This increased fear expression of HAB mice may be caused either by an increased inability to inhibit fear responses, which has also been described in humans [42], or by building a stronger fear memory. Both ideas reflect psychopathology of enhanced fear.

In order to investigate whether short-term memory of conditioned fear is perturbed in HAB mice, we subjected mice of both lines to fear-expression tests within a few hours following cued fear acquisition. While HAB mice always displayed pronounced fear responses to the CS that were similar to the 24 h fear expression, the amount of freezing was significantly reduced in NAB mice 6 h and already 1 h after fear conditioning. This indicates low retention in the memory of the CS-US association following conditioning using the present protocol. However, since conditioned fear expression remains high in CD-1 mice when a much more aversive (10×3 mA foot shock) conditioning protocol is applied [43], it may be further speculated that NAB mice would require stronger fear conditioning for better association of the CS-US.

Since locomotion is reduced in novel environments, but not in the home cage [23], [44] in HAB mice compared with NAB mice, it might be argued that the observed differences in freezing levels in response to a CS may simply reflect altered activity between the two lines. This, however, is unlikely for several reasons. First, when HAB and NAB mice were placed into the conditioning chamber, they showed hardly any freezing behaviour prior presentation of the first US, indicating that the testing contexts per se did not elicit fear responses and, importantly, these low freezing levels did not differ between the two lines. Second, unconditioned animals from both lines did not show any freezing in response to the CS; and third, in both HAB and NAB mice freezing levels increased at a similar rate reaching the same maximum level during fear conditioning.

On the other hand, there is evidence that stress responses are related to an inborn sensitivity to anxiety and pain [45], [46]. For example, there is a high overlap between brain circuitries mediating stress, anxiety and nociception, including those involving the amygdala, the hypothalamus and the neocortex [47], [48]. Interestingly, pain sensitivity is altered in patients with high levels of anxiety or PTSD (e.g. [49], [50], [51], [52], [53]). Despite a proposed dissociation between pain sensitivity and conditioned emotional responses to pain in normal rats [54], it may be speculated that the differences that exist between HAB and NAB mice in terms of fear-memory consolidation are secondary to their differences in sensitivity to the US. In order to investigate this idea, animals were subjected to a flinch/jump test. It was found that the two lines did not differ in terms of the threshold of shock perception as indicated by flinches or jumps expressing emotionally driven reactions in response to the foot shock [55], which suggests that enhanced fear expression in HAB mice is not driven by an altered pain perception upon US exposure. Similar to the HAB mouse line, baseline mechanical pain sensitivity has also been found to be unaltered in HAB rats [56]. Although selective breeding for innate anxiety did not coincide with a selection for innate aversion to painful stimuli at the sensorimotor level (shock reactivity), the possibility cannot be excluded, that differences in the processing and storage of the shock response in HAB mice compared with NAB mice may have contributed to their altered fear memories.

We were further stimulated by studies showing that the circadian clock, known to regulate locomotor activity and arousal of the central nervous system, may also affect learning and memory [41], [57], [58]. Moreover, since there is evidence that hippocampal long-term potentiation, which is a neuronal correlate of learning and memory, is greater and more stable during the night than it is during the daytime [59], [60], we performed fear-conditioning experiments during the night cycle, when both lines are highly active (data not shown). Again, both HAB and NAB mice acquired cued conditioned fear responses to the same extent, while 24 h later, fear expression was more pronounced in HAB mice compared to NAB mice. Freezing levels in response to the CS were comparable between the light and dark phases, indicating that the circadian cycle did not influence fear responses in either line using the described protocol. Thus, it is suggested that fear conditioning in HAB and NAB mice is robust and independent of the activity phase it is tested in. Hence, testing of the fear-reducing potential of compounds in HAB mice (see below) can be reliably performed during daytime, when their inactive phase coincides with general business hours.

Finally, we investigated whether it was possible to attenuate the increased conditioned fear responses of HAB mice by pharmacological interventions. NAB animals were not included into this set of experiments as their fear expression was already very low (see Figures 1 and 2) and, thus, reproducible fear-reducing effects of drugs would have been difficult to detect limiting the additional pharmacological information gained. At first, we tested the effect of the α_2,3,5_-subunit selective BZD partial agonist L-838,417 on fear expression in the HAB line since the attenuation of anxious states by compounds acting at the BZD binding site of the GABA-A receptor is well established both experimentally [18] and clinically [30], [32], [61]. L-838,417, thought to be non-sedative compared with other clinically effective BZDs [62], indeed reduced fear expression in HAB mice. At this stage it should be mentioned that we cannot completely rule out a slight sedative effect of the drug in HAB mice, as indicated by a lesser, yet statistically insignificant, effect in terms of the distance travelled during the phase before CS onset, compared with vehicle-treated controls. However, concerning the most important (i.e. fear reducing) effect of the drug, a potential sedative drug effect would have rather increased freezing levels, which is opposed to the reduction we observed in HAB mice. Possible sedative effects of L-838,417 will be investigated in follow up studies using a range of drug doses and different behavioural tests. To our knowledge, this is the first study demonstrating a reduction of enhanced conditioned fear responses in a psychopathological mouse model of trait anxiety through use of this drug. Furthermore, we have provided the first evidence of pharmacological sensitivity of adult HAB mice to an established class of anxiolytics, the BZDs.

In addition to BZDs, both the NK1 receptor antagonist L-822,429 and CORT suppressed the freezing behaviour displayed by HAB mice during the fear-expression test, with L-822,429 being the most effective. The fear-reducing effect of L-822,429 could be enhanced by chronic treatment. Anxiolytic effects of NK1 receptor antagonists have been demonstrated in rodents and humans after both acute and chronic treatments [28], [63], [64]. Besides, NK1 receptor antagonists are promnestic [65] rendering it unlikely that chronic treatment with L-822,429 impaired fear learning. Indeed, chronic treatment with the NK1 receptor antagonist did not affect fear conditioning in HAB animals per se, further suggesting that fear conditioning and expression of learned fear represent two distinct processes, most likely only the latter involving the NK1 receptor system. The fear-reducing effects of CORT in HAB subjects can be explained by its ability to inhibit the recall of previously acquired, emotionally arousing information [66], [67], [68], [69], [70], [71] – an ability that has so far been demonstrated in “normal” animals [72], [73], [74], [75] and in humans with PTSD or phobias [30], [32], [61]. This effect is opposite of the evidence for an acute anxiogenic effect of CORT in tests of anxiety [76]. The fact that CORT and diverse NK1 receptor antagonists are currently in various stages of clinical trials concerned with the treatment of anxiety disorders [28], [31], [77], [78] underlines a potential translational value to the present results in HAB mice.

Using an experimental paradigm of learned fear in combination with mice selectively bred for either high or normal anxiety-related behaviour, these findings demonstrate an association between innate anxiety and enhanced fear expression. Furthermore, it was shown that the enhanced fear expression in HAB mice could be attenuated by a BZD, a well established anxiolytic class of drugs, as well as by CORT and a NK1 receptor antagonist, which are currently under clinical trials for the treatment of specific anxiety disorders. Thus, the HAB mouse may be a particularly powerful model for the functional analysis of neuroanatomical networks, candidate genes and gene products underlying susceptibility to learned emotionality [79], [80], and for the development of novel, improved pharmacotherapies for patients with fear-associated disorders, including PTSD and phobias.

## Materials and Methods

### Ethics statement

All experiments performed were approved by the Austrian Ethical Committee on Animal Care and Use (Bundesministerium für Bildung, Wissenschaft und Kultur, Austria; approval ID: BMBWK-66.008/0002-BrGT/2007) in line with international laws and policies with the aim of minimizing animal suffering and reducing the number of animals needed.

### Animals

Experiments were carried out on adult male HAB (n = 113) and NAB mice (n = 46) at 12–15 weeks of age bred in the animal facilities of the Department of Pharmacology and Toxicology, University of Innsbruck, Austria. As previously described in more detail [23], the two lines were derived from a Swiss CD-1 outbred population selectively inbred for either high or normal anxiety-related behaviour displayed on the elevated plus maze, with HAB mice spending less than 15% of the testing time on its open arms, compared with approximately 25–35% for NAB mice with no overlapping between the lines. The behavioural phenotype of each mouse was confirmed by an elevated plus maze test at 7 weeks of age. Animals were group housed (up to four litter mates per cage), and supplied with pelleted food and water *ad libitum* under standard laboratory conditions (12 h light/dark cycle with lights on at 7:00, 22±1°C, 60% humidity).

### Fear conditioning

Behavioural experiments were carried out either during the light phase (8:00–15:00) or the dark phase (20:00–3:00) of the cycle as indicated below. All paradigms consisted of both a fear-conditioning session and a fear-expression test at different post conditioning intervals, i.e. presenting the CS to the animals for a short duration.

#### Contextual fear conditioning

Contextual fear conditioning was performed in a fully automated fear-conditioning system (TSE, Technical & Scientific Equipment GmbH, Bad Homburg, Germany) consisting of a Perspex arena (23×23×35 cm) and a metal grid floor. For conditioning, mice were placed into the brightly illuminated (300 lux) context and five unsignalled mild foot shocks (0.7 mA, 2 s; US) were delivered. 2-min stimulus-free periods preceded, separated, and followed the US presentations. 24 h after the fear conditioning, mice were returned to the same context for 3 min to assess fear expression. The arena was thoroughly cleaned with tap water between each animal.

#### Cued auditory fear conditioning

Cued fear conditioning was performed according to a protocol described previously [81]. On day 1, HAB and NAB mice received five pairings (inter-trial interval 2 min) of a tone (white noise, 80 dB, 2 min; CS) with a co-terminating mild foot shock (0.7 mA, 2 s; US) in a clean standard conditioning chamber (26×30×32 cm; Coulbourn Instruments, Allentown, PA, USA) under bright illumination (300 lux). In order to control for unspecific behavioural responses to the CS, separate groups of each line were presented with the CS only, never paired with the US. 24 h later (day 2), mice were placed in a standard empty mouse cage (26×20×13 cm, swiped with ethanol) with a smooth surface and illuminated by dim red light, where 3 CS separated by 5-s intervals were presented. In addition, fear expression was also tested 1 h and 6 h after the conditioning in separate groups of animals. In all sessions, animals were given 2-min stimulus-free periods prior to and post last stimulus exposure. Stimulus presentation was controlled by the Habitest operant system (Coulbourn Instruments, Allentown, USA). Foot shocks were delivered via an interface to the metal grid of the conditioning chamber, and auditory stimuli were applied via a speaker (Coulbourn Instruments, Allentown, PA, USA) installed above the contexts.

#### Behavioural analysis

All sessions were recorded via individual video cameras mounted above each context. Freezing behaviour, defined as the absence of all non-respiratory movements [82], [83], was taken as the measure of fear and was scored by an experienced investigator blinded to mouse lines and treatments. In contextual fear-conditioning experiments percentages of freezing time for each mouse were calculated during each 2-min period post US presentation in the conditioning and during the 3-min context exposure in the fear expression tests while percentages of freezing time for each mouse were calculated during each CS presented in conditioning and during 3 CSs in fear expression sessions in cued auditory fear-conditioning experiments.

### Drug treatments

#### Acute treatments

The α_2,3,5_-subunit selective BZD partial agonist L-838,417 (1 mg/kg; kindly donated by Merck) was suspended in a 0.5% methylcellulose solution (Sigma). CORT (10 mg/kg; Sigma-Aldrich) was dissolved in a 45% cyclodextrine solution (Sigma). The non-peptidergic, brain-penetrating NK1 receptor antagonist L-822,429 [2-cyclopropoxy-5-(5-(trifluoromethyl)tetrazol-1-yl)benzyl]-(2-phenyl-piperidin-3-yl)amine (30 mg/kg), synthesized in house with high affinity to the rat and mouse NK1 receptor [29] was dissolved in saline. Drugs were administered *intra-peritoneally* to HAB mice 30 min prior to the fear-expression test. Control animals received vehicle only.

#### Chronic treatment

The NK1 receptor antagonist L-822,429 was chronically applied to HAB mice via the drinking water for three weeks. A drug intake of approximately 30 mg/kg/day was achieved by adapting the concentrations of the NK1 receptor antagonist in the drinking solutions according to mean drinking volume and body weight per cage. Control HAB mice were supplied with pure tap water.

### Flinch/jump test

Since high freezing levels have been only described in unselected CD-1 mice when foot shocks with high amperages were used [43], separate HAB and NAB groups were tested in the flinch/jump test. Animals were individually placed in standard conditioning boxes (see above). After 2 min of habituation to the chamber, animals were subjected to 1 s shocks of gradually increasing amperage (0.05 mA every 30 s) starting from 0.05 mA. Mice were scored for their first visible response to the shock (flinch), their first pronounced motor response (run or jump), and their first vocalized distress, as previously described [84].

### Data presentation and statistics

Data represent mean ± standard error of the mean (SEM). Statistical analysis was performed using STATISTICA 7.1 (Stat Soft, Inc., USA). First, all data were tested for homoscedasticity using Levene's test. Since a non-parametric distribution was revealed for behavioural measures in the flinch/jump test, data were statistically analyzed using a Mann-Whitney U test. All other data followed a parametric distribution, and thus were further analyzed using a multiple-way ANOVA with repeated measures followed by a Bonferroni test, where allowed. Significant differences in the fear expression test were analyzed using a Student's t-test.

## References

[1] Davis A, Aggleton JP (2000). The role of the amygdala in conditioned and unconditioned fear and anxiety.. The amygdala: A functional analysis.

[2] McEwen BS (2004). Protection and damage from acute and chronic stress: allostasis and allostatic overload and relevance to the pathophysiology of psychiatric disorders.. Ann N Y Acad Sci.

[3] Kessler RC, Berglund P, Demler O, Jin R, Merikangas KR (2005). Lifetime prevalence and age-of-onset distributions of DSM-IV disorders in the National Comorbidity Survey Replication.. Arch Gen Psychiatry.

[4] Kessler RC, Sonnega A, Bromet E, Hughes M, Nelson CB (1995). Posttraumatic stress disorder in the National Comorbidity Survey.. Arch Gen Psychiatry.

[5] Yehuda R (2004). Understanding heterogeneous effects of trauma exposure: relevance to postmortem studies of PTSD.. Psychiatry.

[6] Jovanovic T, Ressler KJ (2010). How the neurocircuitry and genetics of fear inhibition may inform our understanding of PTSD.. Am J Psychiatry.

[7] Mineka S, Oehlberg K (2008). The relevance of recent developments in classical conditioning to understanding the etiology and maintenance of anxiety disorders.. Acta Psychol (Amst).

[8] Chambers JA, Power KG, Durham RC (2004). The relationship between trait vulnerability and anxiety and depressive diagnoses at long-term follow-up of Generalized Anxiety Disorder.. J Anxiety Disord.

[9] Sandi C, Richter-Levin G (2009). From high anxiety trait to depression: a neurocognitive hypothesis.. Trends Neurosci.

[10] Bush DE, Sotres-Bayon F, LeDoux JE (2007). Individual differences in fear: isolating fear reactivity and fear recovery phenotypes.. J Trauma Stress.

[11] Lopez-Aumatell R, Blazquez G, Gil L, Aguilar R, Canete T (2009). The Roman High- and Low-Avoidance rat strains differ in fear-potentiated startle and classical aversive conditioning.. Psicothema.

[12] Lopez-Aumatell R, Vicens-Costa E, Guitart-Masip M, Martinez-Membrives E, Valdar W (2009). Unlearned anxiety predicts learned fear: a comparison among heterogeneous rats and the Roman rat strains.. Behav Brain Res.

[13] Ponder CA, Kliethermes CL, Drew MR, Muller J, Das K (2007). Selection for contextual fear conditioning affects anxiety-like behaviors and gene expression.. Genes Brain Behav.

[14] Fernandez-Teruel A, Escorihuela RM, Gray JA, Aguilar R, Gil L (2002). A quantitative trait locus influencing anxiety in the laboratory rat.. Genome Res.

[15] Brigman JL, Mathur P, Lu L, Williams RW, Holmes A (2009). Genetic relationship between anxiety-related and fear-related behaviors in BXD recombinant inbred mice.. Behav Pharmacol.

[16] Talbot CJ, Radcliffe RA, Fullerton J, Hitzemann R, Wehner JM (2003). Fine scale mapping of a genetic locus for conditioned fear.. Mamm Genome.

[17] Heim C, Nemeroff CB (2009). Neurobiology of posttraumatic stress disorder.. CNS Spectr.

[18] Millan MJ (2003). The neurobiology and control of anxious states.. Prog Neurobiol.

[19] Fendt M, Fanselow MS (1999). The neuroanatomical and neurochemical basis of conditioned fear.. Neurosci Biobehav Rev.

[20] Davis M (1979). Diazepam and flurazepam: effects on conditioned fear as measured with the potentiated startle paradigm.. Psychopharmacology (Berl).

[21] Risbrough VB, Brodkin JD, Geyer MA (2003). GABA-A and 5-HT1A receptor agonists block expression of fear-potentiated startle in mice.. Neuropsychopharmacology.

[22] Santos JM, Gargaro AC, Oliveira AR, Masson S, Brandao ML (2005). Pharmacological dissociation of moderate and high contextual fear as assessed by freezing behavior and fear-potentiated startle.. Eur Neuropsychopharmacol.

[23] Kromer SA, Kessler MS, Milfay D, Birg IN, Bunck M (2005). Identification of glyoxalase-I as a protein marker in a mouse model of extremes in trait anxiety.. J Neurosci.

[24] Marschner A, Kalisch R, Vervliet B, Vansteenwegen D, Buchel C (2008). Dissociable roles for the hippocampus and the amygdala in human cued versus context fear conditioning.. J Neurosci.

[25] Maren S (2008). Pavlovian fear conditioning as a behavioral assay for hippocampus and amygdala function: cautions and caveats.. Eur J Neurosci.

[26] Pavlov IP (1927). Conditioned Reflexes: An Investigation of the Physiological Activity of the Cerebral Cortex..

[27] Watson JB, Rayner R (1920). Conditioned emotional reactions.. J Exp Psychol.

[28] Ebner K, Sartori SB, Singewald N (2009). Tachykinin receptors as therapeutic targets in stress-related disorders.. Curr Pharm Des.

[29] Singewald N, Chicchi GG, Thurner CC, Tsao KL, Spetea M (2008). Modulation of basal and stress-induced amygdaloid substance P release by the potent and selective NK1 receptor antagonist L-822429.. J Neurochem.

[30] Aerni A, Traber R, Hock C, Roozendaal B, Schelling G (2004). Low-dose cortisol for symptoms of posttraumatic stress disorder.. Am J Psychiatry.

[31] Roozendaal B, McEwen BS, Chattarji S (2009). Stress, memory and the amygdala.. Nat Rev Neurosci.

[32] Soravia LM, Heinrichs M, Aerni A, Maroni C, Schelling G (2006). Glucocorticoids reduce phobic fear in humans.. Proc Natl Acad Sci U S A.

[33] McKernan RM, Rosahl TW, Reynolds DS, Sur C, Wafford KA (2000). Sedative but not anxiolytic properties of benzodiazepines are mediated by the GABA(A) receptor alpha1 subtype.. Nat Neurosci.

[34] Borta A, Wohr M, Schwarting RK (2006). Rat ultrasonic vocalization in aversively motivated situations and the role of individual differences in anxiety-related behavior.. Behav Brain Res.

[35] Muigg P, Hetzenauer A, Hauer G, Hauschild M, Gaburro S (2008). Impaired extinction of learned fear in rats selectively bred for high anxiety–evidence of altered neuronal processing in prefrontal-amygdala pathways.. Eur J Neurosci.

[36] Barrett J, Armony JL (2009). Influence of trait anxiety on brain activity during the acquisition and extinction of aversive conditioning.. Psychol Med.

[37] Adams B, Fitch T, Chaney S, Gerlai R (2002). Altered performance characteristics in cognitive tasks: comparison of the albino ICR and CD1 mouse strains.. Behav Brain Res.

[38] Diana G, Valentini G, Travaglione S, Falzano L, Pieri M (2007). Enhancement of learning and memory after activation of cerebral Rho GTPases.. Proc Natl Acad Sci U S A.

[39] Bunck M, Czibere L, Horvath C, Graf C, Frank E (2009). A hypomorphic vasopressin allele prevents anxiety-related behavior.. PLoS One.

[40] Falconer DS, Mackay TFC (1996). Introduction to Quantitative Genetics 4th Ed..

[41] Valentinuzzi VS, Kolker DE, Vitaterna MH, Shimomura K, Whiteley A (1998). Automated measurement of mouse freezing behavior and its use for quantitative trait locus analysis of contextual fear conditioning in (BALB/cJ x C57BL/6J)F2 mice.. Learn Mem.

[42] Lissek S, Powers AS, McClure EB, Phelps EA, Woldehawariat G (2005). Classical fear conditioning in the anxiety disorders: a meta-analysis.. Behav Res Ther.

[43] Mikics E, Dombi T, Barsvari B, Varga B, Ledent C (2006). The effects of cannabinoids on contextual conditioned fear in CB1 knockout and CD1 mice.. Behav Pharmacol.

[44] Muigg P, Scheiber S, Salchner P, Bunck M, Landgraf R (2009). Differential stress-induced neuronal activation patterns in mouse lines selectively bred for high, normal or low anxiety.. PLoS One.

[45] Burns JW (2006). The role of attentional strategies in moderating links between acute pain induction and subsequent psychological stress: evidence for symptom-specific reactivity among patients with chronic pain versus healthy nonpatients.. Emotion.

[46] Xu K, Ernst M, Goldman D (2006). Imaging genomics applied to anxiety, stress response, and resiliency.. Neuroinformatics.

[47] LeDoux JE (2000). Emotion circuits in the brain.. Annu Rev Neurosci.

[48] Neugebauer V, Li W, Bird GC, Han JS (2004). The amygdala and persistent pain.. Neuroscientist.

[49] Asmundson GJ, Norton GR (1995). Anxiety sensitivity in patients with physically unexplained chronic back pain: a preliminary report.. Behav Res Ther.

[50] Defrin R, Ginzburg K, Solomon Z, Polad E, Bloch M (2008). Quantitative testing of pain perception in subjects with PTSD–implications for the mechanism of the coexistence between PTSD and chronic pain.. Pain.

[51] Keogh E, Mansoor L (2001). Investigating the effects of anxiety sensitivity and coping on the perception of cold pressor pain in healthy women.. Eur J Pain.

[52] Kraus A, Geuze E, Schmahl C, Greffrath W, Treede RD (2009). Differentiation of pain ratings in combat-related posttraumatic stress disorder.. Pain.

[53] Pitman RK, van der Kolk BA, Orr SP, Greenberg MS (1990). Naloxone-reversible analgesic response to combat-related stimuli in posttraumatic stress disorder. A pilot study.. Arch Gen Psychiatry.

[54] Lehner M, Wislowska-Stanek A, Maciejak P, Szyndler J, Sobolewska A (2010). The relationship between pain sensitivity and conditioned fear response in rats.. Acta Neurobiol Exp (Wars).

[55] Evans WO (1961). A new technique for the investigation of some analgesic drugs on a reflexive behavior in the rat.. Psychopharmacologia.

[56] Roeska K, Ceci A, Treede RD, Doods H (2009). Effect of high trait anxiety on mechanical hypersensitivity in male rats.. Neurosci Lett.

[57] Chaudhury D, Colwell CS (2002). Circadian modulation of learning and memory in fear-conditioned mice.. Behav Brain Res.

[58] Fernandez RI, Lyons LC, Levenson J, Khabour O, Eskin A (2003). Circadian modulation of long-term sensitization in Aplysia.. Proc Natl Acad Sci U S A.

[59] Barnes CA, McNaughton BL, Goddard GV, Douglas RM, Adamec R (1977). Circadian rhythm of synaptic excitability in rat and monkey central nervous system.. Science.

[60] Chaudhury D, Wang LM, Colwell CS (2005). Circadian regulation of hippocampal long-term potentiation.. J Biol Rhythms.

[61] Bentz D, Michael T, de Quervain DJ, Wilhelm FH (2010). Enhancing exposure therapy for anxiety disorders with glucocorticoids: from basic mechanisms of emotional learning to clinical applications.. J Anxiety Disord.

[62] Atack JR (2003). Anxioselective compounds acting at the GABA(A) receptor benzodiazepine binding site.. Curr Drug Targets CNS Neurol Disord.

[63] Ebner K, Singewald N (2006). The role of substance P in stress and anxiety responses.. Amino Acids.

[64] Czeh B, Fuchs E, Simon M (2006). NK1 receptor antagonists under investigation for the treatment of affective disorders.. Expert Opin Investig Drugs.

[65] Kart-Teke E, Dere E, Brandao ML, Huston JP, De Souza Silva MA (2007). Reinstatement of episodic-like memory in rats by neurokinin-1 receptor antagonism.. Neurobiol Learn Mem.

[66] de Quervain DJ, Henke K, Aerni A, Treyer V, McGaugh JL (2003). Glucocorticoid-induced impairment of declarative memory retrieval is associated with reduced blood flow in the medial temporal lobe.. Eur J Neurosci.

[67] de Quervain DJ, Roozendaal B, McGaugh JL (1998). Stress and glucocorticoids impair retrieval of long-term spatial memory.. Nature.

[68] de Quervain DJ, Roozendaal B, Nitsch RM, McGaugh JL, Hock C (2000). Acute cortisone administration impairs retrieval of long-term declarative memory in humans.. Nat Neurosci.

[69] Roozendaal B, Griffith QK, Buranday J, De Quervain DJ, McGaugh JL (2003). The hippocampus mediates glucocorticoid-induced impairment of spatial memory retrieval: dependence on the basolateral amygdala.. Proc Natl Acad Sci U S A.

[70] Roozendaal B, Hahn EL, Nathan SV, de Quervain DJ, McGaugh JL (2004). Glucocorticoid effects on memory retrieval require concurrent noradrenergic activity in the hippocampus and basolateral amygdala.. J Neurosci.

[71] Wolf OT, Kuhlmann S, Buss C, Hellhammer DH, Kirschbaum C (2004). Cortisol and memory retrieval in humans: influence of emotional valence.. Ann N Y Acad Sci.

[72] Roozendaal B, de Quervain DJ, Schelling G, McGaugh JL (2004). A systemically administered beta-adrenoceptor antagonist blocks corticosterone-induced impairment of contextual memory retrieval in rats.. Neurobiol Learn Mem.

[73] Skorzewska A, Bidzinski A, Lehner M, Turzynska D, Sobolewska A (2007). The effects of acute corticosterone administration on anxiety, endogenous corticosterone, and c-Fos expression in the rat brain.. Horm Behav.

[74] Skorzewska A, Bidzinski A, Lehner M, Turzynska D, Wislowska-Stanek A (2006). The effects of acute and chronic administration of corticosterone on rat behavior in two models of fear responses, plasma corticosterone concentration, and c-Fos expression in the brain structures.. Pharmacol Biochem Behav.

[75] Thompson BL, Erickson K, Schulkin J, Rosen JB (2004). Corticosterone facilitates retention of contextually conditioned fear and increases CRH mRNA expression in the amygdala.. Behav Brain Res.

[76] Mitra R, Sapolsky RM (2008). Acute corticosterone treatment is sufficient to induce anxiety and amygdaloid dendritic hypertrophy.. Proc Natl Acad Sci U S A.

[77] ClinicalTrials.gov website (accessed 2010) http://clinicaltrials.gov/ct2/results?term= cortisol+anxiety

[78] ClinicalTrials.gov website (accessed 2010) http://clinicaltrials.gov/ct2/results?term= neurokinin+anxiety

[79] Pape HC, Pare D (2010). Plastic synaptic networks of the amygdala for the acquisition, expression, and extinction of conditioned fear.. Physiol Rev.

[80] Pape HC, Stork O (2003). Genes and mechanisms in the amygdala involved in the formation of fear memory.. Ann N Y Acad Sci.

[81] Busquet P, Hetzenauer A, Sinnegger-Brauns MJ, Striessnig J, Singewald N (2008). Role of L-type Ca2+ channel isoforms in the extinction of conditioned fear.. Learn Mem.

[82] Blanchard RJ, Blanchard DC (1969). Passive and active reactions to fear-eliciting stimuli.. J Comp Physiol Psychol.

[83] Fanselow MS (1980). Conditioned and unconditional components of post-shock freezing.. Pavlov J Biol Sci.

[84] Wittmann W, Schunk E, Rosskothen I, Gaburro S, Singewald N (2009). Prodynorphin-derived peptides are critical modulators of anxiety and regulate neurochemistry and corticosterone.. Neuropsychopharmacology.

